# Structural Insights into Complexes of Glucose-Regulated Protein94 (Grp94) with Human Immunoglobulin G. Relevance for Grp94-IgG Complexes that Form *In Vivo* in Pathological Conditions

**DOI:** 10.1371/journal.pone.0086198

**Published:** 2014-01-28

**Authors:** Andrea Pagetta, Elisa Tramentozzi, Elena Tibaldi, Laura Cendron, Giuseppe Zanotti, Anna Maria Brunati, Maurizio Vitadello, Luisa Gorza, Paola Finotti

**Affiliations:** 1 Department of Pharmaceutical and Pharmacological Sciences, University of Padova, Padova, Italy; 2 Department of Molecular Medicine, University of Padova, Padova, Italy; 3 Department of Biomedical Sciences, University of Padova, Padova, Italy; 4 Centro Nazionale delle Ricerche, CNR - Institute of Neurosciences, Padova Section, Padova, Italy; University of Quebect at Trois-Rivieres, Canada

## Abstract

While the mechanism by which Grp94 displays its chaperone function with client peptides in the cell has been elucidated extensively, much less is known about the nature and properties of how Grp94 can engage binding to proteins once it is exposed on the cell surface or liberated in the extra-cellular milieu, as occurs in pathological conditions.

In this work, we wanted to investigate the molecular aspects and structural characteristics of complexes that Grp94 forms with human IgG, posing the attention on the influence that glycosylation of Grp94 might have on the binding capacity to IgG, and on the identification of sites involved in the binding. To this aim, we employed both native, fully glycosylated and partially glycosylated Grp94, and recombinant, non-glycosylated Grp94, as well as IgG subunits, in different experimental conditions, including the physiological setting of human plasma. Regardless of the species and type, Grp94 engages a similar, highly specific and stable binding with IgG that involves sites located in the N-terminal domain of Grp94 and the hinge region of whole IgG. Grp94 does not form stable complex with Fab, F(ab)_2_ or Fc. Glycosylation turns out to be an obstacle to the Grp94 binding to IgG, although this negative effect can be counteracted by ATP and spontaneously also disappears in time in a physiological setting of incubation. ATP does not affect at all the binding capacity of non-glycosylated Grp94. However, complexes that native, partially glycosylated Grp94 forms with IgG in the presence of ATP show strikingly different characteristics with respect to those formed in absence of ATP.

Results have relevance for the mechanism regulating the formation of stable Grp94-IgG complexes *in vivo*, in the pathological conditions associated with the extra-cellular location of Grp94.

## Introduction

The main endoplasmic reticulum (ER)-resident Heat Shock Protein (HSP) Glucose-regulated protein94 (Grp94) is unique among HSPs since, besides the chaperone function it also fulfills a complex immuno-modulatory activity [1]–[3]. Grp94 also differs from other HSPs, including its cytoplasmic paralog HSP90, in the number and type of client proteins [4], [5], mechanism of folding [6] and assistance by other co-chaperones [7], [8]. While the mechanism by which Grp94 displays the chaperone activity has been investigated in detail [9]–[11], and a wealth of experimental evidence exists to demonstrate that Grp94 binds to peptides *in vitro* [12]–[15], much less is known about Grp94 binding to proteins [16], in particular in the extra-cellular setting. This aspect is of particular relevance in the light of the properties acquired by Grp94 in the un-physiological extra-cellular location where it is always sensed as an immunological danger capable of triggering intense immune reactions [17], [18]. The expression of Grp94 on the cell membrane has been observed to cause the development of autoimmune disease in experimental animals [19]. Similarly, the increase in the plasma concentration of both Grp94 and anti-Grp94 antibodies (Abs) found in various auto-immune/inflammatory conditions [19]–[23] has been taken as a proof of the role played by Grp94 in the pathogenesis of these diseases.

In *ex vivo* experiments on plasma of type 1 diabetic subjects we observed that Grp94, besides being present at a higher-than-normal concentration [23], circulated only linked to plasma proteins, mostly IgG, forming complexes of various masses prevalently immune in nature [21], [24]. We further demonstrated that Grp94 could also bind to IgG irrespective of their immune nature, forming non-immune complexes (NICs) in which binding occurs at sites other than the antigen-binding site [25]. These results raised the possibility that NICs might also be present *in vivo*, representing the earliest form of complexes following the exposure on the cell surface and/or liberation of Grp94 in the extra-cellular space. Furthermore, given the stability of the binding in NICs, it was hypothesized that these complexes might acquire an immunogenic potential, causing a further worsening and spreading of the immune reactions that characterize the diabetic disease [25].

Overall, results accumulated so far indicated that binding of Grp94 to circulating IgG has characteristics profoundly different from those displayed by ER-resident Grp94 [11], [26]. Whereas a reversible binding is predicted to occur in chaperoning peptides/proteins in the intra-cellular setting, irreversibility appears to characterize the extra-cellular binding of Grp94 to IgG. The additional observation that Grp94 identified in the complexes circulating in the plasma of diabetic subjects was not glycosylated [20], raised the question of whether this post-transcriptional modification had any relevance in determining the extra-cellular exposure of Grp94 and/or in conferring on Grp94 a peculiar binding stability.

To shed light on the intriguing and still unresolved questions about the complexes that Grp94 might form with IgG in the extra-cellular setting, we present a work in which the nature of Grp94 binding to human pre-immune IgG is investigated in detail in different experimental conditions, using both native rat Grp94, in its fully glycosylated and partially glycosylated forms, and a recombinant, non-glycosylated form of Grp94.

Results support the conclusion that a highly specific and stable binding forms between Grp94 and IgG that involves sites located in the N-terminal domain of Grp94 and in the hinge region of IgG. While non-glycosylated Grp94 binds to IgG in absence of ATP, ATP appears necessary to counteract the negative effect of glycosylation on the binding capacity to IgG, and complexes formed in the presence of ATP show structure and dimensions different from those observed in absence of ATP. However, glycosylation of Grp94 does not appear to hamper the capacity to form stable complexes with IgG in physiological settings and at longer incubation times.

## Materials and Methods

### Materials

The complete list of materials is in (Materials S1 in File S1). Primary Abs used in Western blot analyses were: anti-Grp94 rat monoclonal, anti-calnexin and anti-calreticulin rabbit polyclonal and anti-Grp78 goat polyclonal (Santa Cruz Biotechnology, Santa Cruz, CA, USA); anti-Grp94 rabbit polyclonal (Enzo Life Sciences, Lausen, Switzerland). Secondary Abs were: anti-rabbit and anti-rat IgG biotin-conjugated (Vector Laboratories, Burlingame, CA, USA), anti-goat IgG alkaline phosphatase-conjugated (Santa Cruz Biotechnology) and extravidin alkaline phosphatase-conjugated (Sigma-Aldrich). Novex alkaline phosphatase chemiluminescent substrate was from Invitrogen. All other reagents were of the highest purity grade.

### Ethics Statement

Experiments involving the use of rat livers were made in accordance with NIH guidelines for the Care and Use of Laboratory Animals also approved by the Italian Ministry of Health. Rats were purchased from Charles River Laboratories and housed in the Animal Research Facility of the Department of Molecular Medicine. Rats were maintained under a 12-hour light-dark cycle and given rat chow and water ad libitum. They were sacrificed via exposure to CO_2_. The study design was approved by the Ethics Committee of the University of Padova for the care and use of laboratory animals.

Experiments using human blood samples were performed following the guidelines established by the Italian Agency of Drugs (AIFA) for observational studies and published in the Gazzetta Ufficiale della Repubblica Italiana (31 March 2008). The national low considers that blood samples taken occasionally do not need the approval by the Ethics Committee (GU 31 March 2008, p74). However, before taking blood samples, we consulted our local Ethics Committee. The healthy volunteers were recruited among regular blood donors at the Transfusion Center of the local Hospital (Padova), and they gave their verbal informed consent for having a blood sample specifically taken for this study. The Medical Doctor (FPP) who drew the blood and one of the co-authors (ET) can attest the donors' verbal consent.

### Purification of native Grp94

Native Grp94 was purified from the rat hepatocyte microsomal fraction through multiple chromatographic steps following the method of Lasa et al. [27] (details in Methods S1 in File S1). The microsomal fraction was solubilized in HEPES buffer (pH = 7.0), centrifuged and the supernatant subjected to a DEAE-Sepharose column (Pharmacia, Uppsala, Sweden). A linear (0 to 0.6 M) NaCl gradient was applied for protein elution and fractions immune-reactive with anti-Grp94 Abs were pooled and passed through a Heparin-Sepharose column. Proteins eluted with a linear (from 0 to 0.8 M) gradient of NaCl and the Grp94-containing fractions (0.5 M NaCl) were then chromatographed on a HiLoad 26/60 Superdex 200 prep-grade column (26 mm×600 mm, Pharmacia). Elution was at the flow rate of 0.75 ml/min, and every fourth fraction (1.5 ml each) was tested for Grp94.

Fractions containing purified Grp94 were dialyzed in Slide-A-Lyzer cassettes (3,500 kDa MWCO) (Pierce, Rockford, IL, USA) at room temperature against a 500-fold volume of 10 mM Tris-HCl, pH = 7.0. When necessary, Grp94 solutions were also concentrated in the same Slyde-A-Lyzer cassettes immersed in Slide-A-Lyzer concentrating solution. Proteins were measured both at 280 nm, using the extinction coefficient of E_280_ = 0.884 for a 1-mg/ml solution and a path length of 1 cm [9], and with the micro-BCA method (Pierce, Rockford, IL, USA). Dialyzed Grp94 was stored at −20°C in 50-µl aliquots ready to use.

In separate experiments, the Grp94-containing fractions eluted from the Heparin-Sepharose column were pooled and loaded (4.5 mg proteins) to the Con-A Sepharose 4B column (1 ml, HiTrap™ GE Healthcare Bio-Sciences AB, Uppsala, Sweden) after they were submitted to dialysis and re-suspended in the same buffer used for equilibrating the column (20 mM Tris-HCl, 0.5 M NaCl, 1 mM CaCl_2_, 1 mM MgCl_2_, pH = 7.4). Proteins were eluted at the flow rate of 0.2 ml/min by monitoring the absorbance at 280 nm. Grp94 bound to the column (Con-A Grp94) was eluted by applying 0.5 M methyl α-D-mannopyranoside to the buffer (as above). Fractions from the Con-A column were submitted to ultra-filtration (Amicon ultra centrifugal filter device, 30 kDa MWCO, Millipore, Bellerica, MA, USA) to exchange the buffer (10 mM Tris-HCl, pH = 7.0). The concentration of Con-A Grp94 was 1.3 mg/ml, as determined by measuring absorbance at 280 nm with the extinction coefficient of E_280_ = 0.884.

### Recombinant Grp94

Recombinant rabbit Grp94, both in its almost complete form and in the N- and C-terminal fragments, was prepared in bacteria following previously described procedures [28] (details in Methods S2 in File S1).

The expression of recombinant polypeptides was induced in transformed *E. coli* strain M15 by 2 mM isopropyl-β D-thiogalactoside. Purification of the polypeptides was obtained by affinity chromatography on a Ni^+^-Sepharose column (Qiagen) in the presence of 8 M urea and 10 mM 2-β-mercaptoethanol to avoid the formation of disulfide bonds. After elution, the proteins were dialyzed in a Slyde-A-Lyzer cassette (3,500 MWCO, Pierce) overnight at +4°C against a 500-fold volume of buffer (adapted to our purpose from that described in [29]) containing 50 mM Tris-HCl (pH = 7.5), 500 mM NaCl, 5% (v/v) glycerol and 0.5 µM 2-β-mercaptoethanol. A further dialysis step of 4 h was performed at room temperature against a 200-fold volume of 10 mM Tris-HCl (pH = 7.0) to remove re-naturing buffer.

### Incubation of Grp94 with human IgG to form Grp94-IgG complexes

To obtain complexes of Grp94-IgG, we used human pre-immune IgG (Sigma-Aldrich) the purity of which was preliminarily assessed as described [25] and the protein concentration determined at 280 nm using E_280_ = 1.45 for a 1-mg/ml and a path length of 1 cm. Native rat Grp94 (0.1 mg/ml, final concentration) was incubated at 37°C for 1, 2, 4 and 6 h, with 0.07, 0.15, 0.30, 0.45 mg/ml IgG (corresponding to the Grp94∶IgG molar ratios of 1∶0.5, 1∶1, 1∶2 and 1∶3, if Grp94 is considered in its monomeric form of about 100 kDa and IgG with a molecular mass of 150 kDa). Incubations were performed in a final volume of 100 µl in 10 mM Tris (pH = 7.0) in both absence and presence of 150 mM NaCl. Control solutions of both Grp94 and IgG alone were also incubated separately. In experiments in which recombinant rabbit and native Con-A Grp94 were used to form complexes with IgG, IgG were employed at the concentrations corresponding to the Grp94-IgG molar ratios of 1∶1 and 1∶2, and incubation conducted for 2 h at 37°C, unless otherwise specified.

In experiments of incubation of native Grp94 with human Fab, Fc (Bethyl Laboratories, Inc., Montgomery, TX, USA) and Fab_2_ (Jackson Immuno Research Laboratories Inc., Baltimora, PA, USA), Fab and Fc were used at the final concentrations of 0.05, 0.1 mg/ml, and Fab_2_ at the concentrations of 0.1 and 0.2 mg/ml. Incubation was then conducted at 37°C for 2 h (all other experimental conditions were as those described for integer IgG).

ATP was used at the final concentration of 1 mM and was either pre-incubated with Grp94 at 25°C for 15 min before the addition of IgG (or Fab, Fab_2_ and Fc), or added simultaneously with Grp94 to the IgG (or Fab, Fab_2_ and Fc) solution, followed by incubation at 37°C at the indicated times (see legends to related figures).

### Electrophoresis and Western blot analysis

SDS-PAGE was run on 10% polyacrylamide gel (unless otherwise stated), and gels were stained with standard Coomassie brilliant blue. Unless specified otherwise, 2.5 µg of sample proteins were loaded in any lane, in conditions specified in the legend to the corresponding figures.

For native PAGE, 8.0% and 4.0% polyacrylamide were used for resolving and stacking gels, respectively, prepared with 50 mM Tris-HCl (final concentration) at pH = 8.0, without SDS. A Tris-HCl solution (200 mM, pH = 8.0) was used as running buffer. Sample proteins (2–3 µg, 25–30 µl) were loaded without any further treatment other than the addition of 5–7 µl glycerol (15% final concentration) and trace amount of Orange-G as tracking dye. The run was conducted at the constant voltage of 100 V for 2 h, and the gel then stained with Coomassie blue.

In the 2D-PAGE analysis, samples were first submitted to native PAGE in duplicate lanes of which one served as control and the other was excised and immersed in denaturing solution (0.125 M Tris-HCl, pH 6.8, 4% SDS and trace amount of Orange-G as tracking dye) for 1 h under agitation. Then, it was layered on the second dimension SDS gel (10% polyacrylamide) and the run conducted for the first 20 min at the constant current of 15 mA, to favor electro-elution of samples from the first-dimension lane, and then at 30 mA until completion of the run (for further 45 min).

Western blotting was performed on both SDS- and native PAGE using the primary Abs indicated in the legends to related figures, and either alkaline phosphatase-conjugated, affinity-purified IgG, or biotin-conjugated affinity-purified IgG for the immune detection. An affinity-purified egg white avidin conjugated to alkaline phosphatase (Sigma-Aldrich) was employed for detecting the immune reaction with the ABC system. Secondary Abs alone were used as control to exclude any false positive reaction. The chemi-luminescent reaction was monitored using Molecular Imager® VersaDoc™ MP 4000 System (Bio-Rad, Hemel Hempstead, UK).

### Incubation of native and recombinant Grp94 with human plasma

Plasma was obtained from four healthy volunteers after centrifugation of freshly drawn heparinized blood (10 ml) and submitted to dialysis (Spectra-Por membrane, 15,000 MWCO, A.H. Thomas Philadelphia, PA, USA). Proteins were measured spectrophotometrically and a plasma pool was formed with an equal contribution of protein from each subject. To obtain plasma deprived of IgG, 75 mg of pooled plasma proteins were diluted in phosphate buffer (20 mM, pH = 7.0) and loaded on to a 1-ml HiTrap PG HP column (Amersham Biosciences). Proteins eluted at a flow rate of 0.5 ml/min, and IgG-deprived plasma proteins were recovered in the flow-through by monitoring absorbance at 280 nm. Protein concentration in the eluate was determined using the micro BCA protein assay (Pierce, Rockford, IL, USA).

Grp94 (2.5 µg, both Con-A native and recombinant rabbit Grp94) was incubated with both whole plasma (3.75 mg/ml proteins) and IgG-deprived plasma (3.23 mg/ml proteins, containing the same HSA concentration as the whole plasma) in 500 µl (final volume), with and without 1 mM ATP, for 6.0 and 18 h at 37°C. After incubation, a 10-µl aliquot of each sample was processed in SDS-PAGE on a 4–20% gradient gel (Life Technologies, Carlsbad, CA, USA) followed by Western blotting as specified in the legend to related figures.

### Dynamic Light Scattering

Measurements of dynamic light scattering (DLS) were performed on a Zetasizer Nano ZS (Malvern Instruments Ltd, Malvern, UK), according to the manufacturer's instructions for solvent and acquisition parameters, using a low volume glass cuvette. Each measurement comprised 3 different acquisitions (each being the average of 14 independent accumulations).

Solutions of native Grp94 (0.1 mg/ml), with and without IgG (0.3 mg/ml), were prepared fresh in 10 mM Tris-HCl, pH 7.0, previously filtered through a 0.2 µm pore filter (Millex, Millipore) to prevent dust contamination. Solutions were then incubated at 37°C and DLS measurements made at time intervals of 30 min over 2 h.

### Glycerol density gradient centrifugation

Control solutions of both native Grp94 (0.2 mg/ml) and IgG were incubated (for 2 h at 37°C) both alone and after being mixed together at the 1∶2, Grp94∶IgG molar ratio, and a 240-µl aliquot of any incubated sample (0.1 mg proteins) were subjected to glycerol density gradient centrifugation with 10–40% glycerol in 25 mM Hepes buffer (pH = 7.4) with 1 mM EDTA and 1 mM dithiothreitol. After centrifugation at 100,000×*g* for 18 h at +4°C, the gradient was separated into 18 fractions of 200 µl each and submitted to Western blot analysis for both Grp94 and IgG. Calibration proteins were used for estimating the molecular mass of the complex (see legend to the related Figure).

### Proteolytic digestion of Grp94-IgG complex

Digestion of the Grp94-IgG complex and of native Grp94 and IgG as controls, was performed with papain (10 mg/ml, crystalline suspension in water) used without preliminary activation with cysteine, at the final weight concentration ratios with sample proteins of 1∶100 and 1∶50 (Tris-HCl buffer, pH = 7.0). Samples were incubated at 37°C for 0.5, 1.0, 1.5, 2.0, 4.0, 6.0, 8.0, 12 and 16 h. At any incubation time lyophilized aliquots (25 µl) of each sample were re-suspended with denaturing sample buffer (without reducing agents and boiling) and processed in SDS-PAGE followed by Western blotting.

### Electron microscopy analysis

For electron microscopy measurements, solutions of complexes formed by native Grp94 with IgG together with control native Grp94 and human IgG alone were used at the final protein concentration of 0.06 mg/ml. An aliquot of each sample was absorbed onto glow-discharged carbon-coated butvar films on 400-mesh copper grids. The grids were negatively stained with an un-buffered solution of 1% uranyl acetate, and observed at the microscope (Tecnai G12, Fei Company, Eindhoven, Holland). For each sample, several pictures were taken in separate sections of the grid and those representative of at least two measurements performed on different occasions were presented.

## Results

### Assessment of the Grp94-IgG complex formation in native PAGE. Effects of ATP

**Figure 1:**
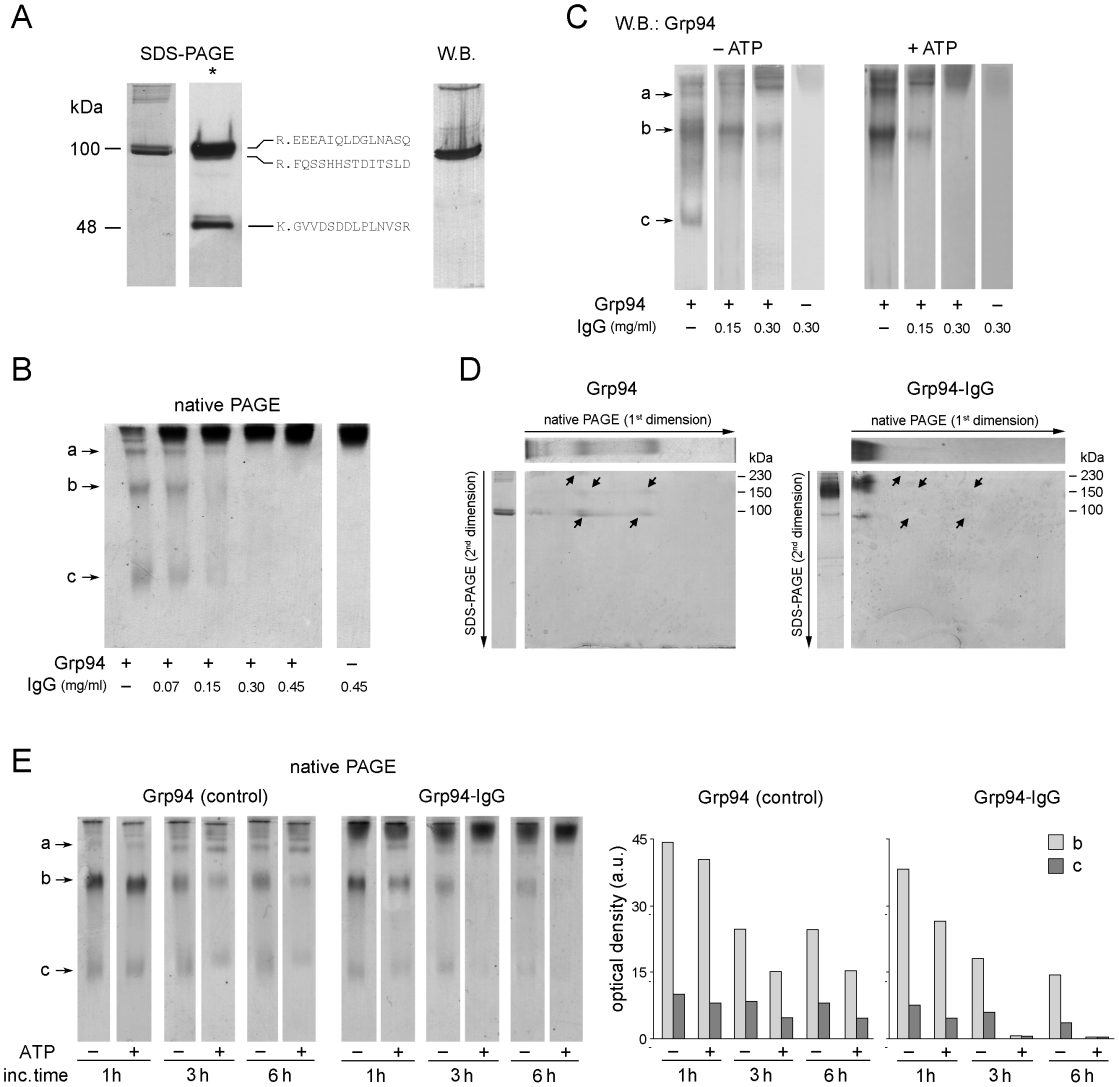
Characteristics of the complex formed by native rat Grp94 with IgG in native conditions. (**A**) A representative fraction of peak 2 from gel filtration was analyzed in SDS-PAGE (8.5% polyacrylamide gel), in both non-reducing and reducing conditions (*) of samples (3 µg proteins/lane). Mass analysis (MALDI-TOF-TOF) for amino-acid sequence determinations was applied to bands (resolved in SDS-PAGE in reducing conditions) after controlled tryptic digestion. The more frequent peptide sequences of each band are indicated on right of the lane, whereas Western blotting (monoclonal anti-Grp94 Abs) is performed on the same fraction following SDS-PAGE in non-reducing conditions. (**B**) Native Grp94 of peak 2 (0.1 mg/ml, 10 mM Tris, pH 7.0) was incubated at 37°C for 120 min in absence of ATP, both alone (control) and with IgG at the indicated concentrations. Three µg of Grp94 were analyzed in native PAGE (8% acrylamide, pH 8.0) and gels stained with Coomassie blue. Lane on right, control IgG at the highest concentration. a, b and c: Grp94 bands with increasing electrophoretic mobility. (**C**) Western blotting for Grp94 on samples as in (B) processed in native PAGE in both absence and presence of ATP (1 mM) preincubated with Grp94 for 15 min before the addition of IgG. (**D**) 2D-PAGE of Grp94 alone (control) and with IgG (at the Grp94∶IgG molar ratio of 1∶2) after incubation at 37°C for 120 min in the presence of ATP, following native PAGE of samples, as in (B) and (C). The lanes of Grp94 alone (3 µg) and with IgG were cut and submitted to the second dimension on 10% acrylamide gel (see Methods). Above and on the left of each gel are lanes of reference of the first dimension and of SDS-PAGE (in non-reducing conditions), respectively. Long arrows indicate the direction of the run (from the cathode to the anode) in both PAGEs, whereas short arrows in the gels mark Grp94 bands that disappear after co-incubation with IgG (gel on right). (**E**) Native PAGE of Grp94 incubated with 0.3 mg/ml IgG and processed as in B at the indicated incubation times, in both absence and presence of ATP (as in C). On right: histograms representing the densitometric analysis of bands b and c of each lane (Gel-Pro Analyzer software, version 3.1). Heights of histograms indicate the optical density (in arbitrary units).

In the last step of purification from rat liver microsomes, gel filtration permitted us to separate different sized species of Grp94 (Figure S1A in File S1). SDS-resistant aggregates of Grp94 were the exclusive species present in peak 1 with an apparent molecular weight of 700 kDa consistent with the non-globular shape of Grp94 (Figure S1B in File S1). Grp94 eluted in peak 2 of gel filtration was apparently pure and was detected in two closely spaced bands at ≈100 kDa, and in one band at 48 kDa, more evident after reducing treatment of samples in SDS-PAGE (Figure 1A). Mass analysis identified full-length Grp94 in the two ≈100 kDa bands, whereas the 48-kDa band corresponded to the C-terminal portion (from the Lys_384_) of Grp94 (Figure 1A). This short form of Grp94, that eluted as a single form of Grp94 in peak 3 of gel filtration (Figure S1B in File S1), did not appear to be a proteolytic product of the full-length protein, since it was already present in the microsomal fraction of hepatocytes before the first step of purification (data not shown). Purity of Grp94 in peak 2 was also assessed by excluding the presence of other ER-resident chaperones that eluted separately in both peak 1 (calnexin) and peak 3 (Grp78 and calreticulin) (Figure S1B in File S1). For these reasons, pure Grp94 eluted in fractions of peak 2 was used in following experiments for testing the formation of complexes with IgG.

To have a reliable information about the species of complexes that might form in physiological conditions, we first analyzed samples in native PAGE. To this aim, Grp94 (0.1 mg/ml) was incubated with IgG at the Grp94∶IgG molar ratios of 1∶0.5, 1∶1, 1∶2 and 1∶3 for 0, 1 and 2 h at 37°C. Control Grp94 focused in three bands with distinct electrophoretic mobility (Figure 1B: “a”, “b” and “c” from the cathode to the anode), that did not change in intensity and mobility at longer incubation times. After incubation with IgG, especially at the Grp94∶IgG molar ratio of 1∶2 and after incubation of 2 h, Grp94 bands disappeared (Figure 1B). The same effect was observed in the presence of 0.15 M NaCl (data not shown). Western blot analysis made on samples previously submitted to native PAGE confirmed the shift of Grp94 to the cathode in bands overlapping that of IgG (Figure 1C, left panel), thus suggesting the formation of complexes. However, since a faint band of Grp94 (band “b”) was still visible after incubation with IgG (Figure 1C, left panel), the possibility was considered that part of Grp94 dissociated from the complex or was unable to bind to IgG, remaining free in solution.

Since Grp94, likewise other HSP90 chaperones, adopts significantly different conformations following ATP binding [6], [11], [30], [31]; and since changes in ATP-driven conformation of these HSPs influence chaperone activity [9], we investigated whether ATP had any effect on species of native Grp94 that apparently did not bind to IgG. In the presence of ATP (1.0 mM, final concentration), bands referred to free Grp94 completely disappeared and all Grp94 was detected in the band of IgG, suggesting that every species of Grp94 was engaged in binding to IgG (Figure 1C, right panel). To further confirm that in the presence of ATP Grp94 was fully sequestered into a complex with IgG, a second dimension SDS-PAGE in non-reducing conditions was performed following native PAGE to compare the pattern of Grp94 before and after incubation with IgG. Whereas control Grp94 in bands of different mobility in native PAGE was resolved into the single species of the monomer (and only partially dimer) in the second dimension (Figure 1D, left panel), none of the Grp94 bands were instead visible in the second dimension after incubation with IgG (Figure 1D, right panel, arrows), and Grp94 was entirely confined into the band of IgG (left side of the gel) from which it partially dissociated as monomer due to SDS.

To better understand what was the effect of ATP on the complex formation, we conducted experiments in which Grp94 was incubated at 37°C for 1.0, 3.0 and 6.0 h with and without IgG, in both absence and presence of ATP, and mobility followed in native PAGE (Figure 1E). ATP increased the rate of the aggregating tendency of Grp94 that spontaneously occurred in time (Figure 1E, left panel, band “a”), and at any incubation time this effect turned out to enhance the rapid and complete disappearance of free Grp94 observed in the presence of IgG (Figure 1E, right panel and densitometric analysis). Results indicated that in the presence of ATP also the species of Grp94 that in absence of ATP did not bind to IgG apparently adopted a conformation that favored binding.

### Direct demonstration of the Grp94-IgG complex formation

**Figure 2:**
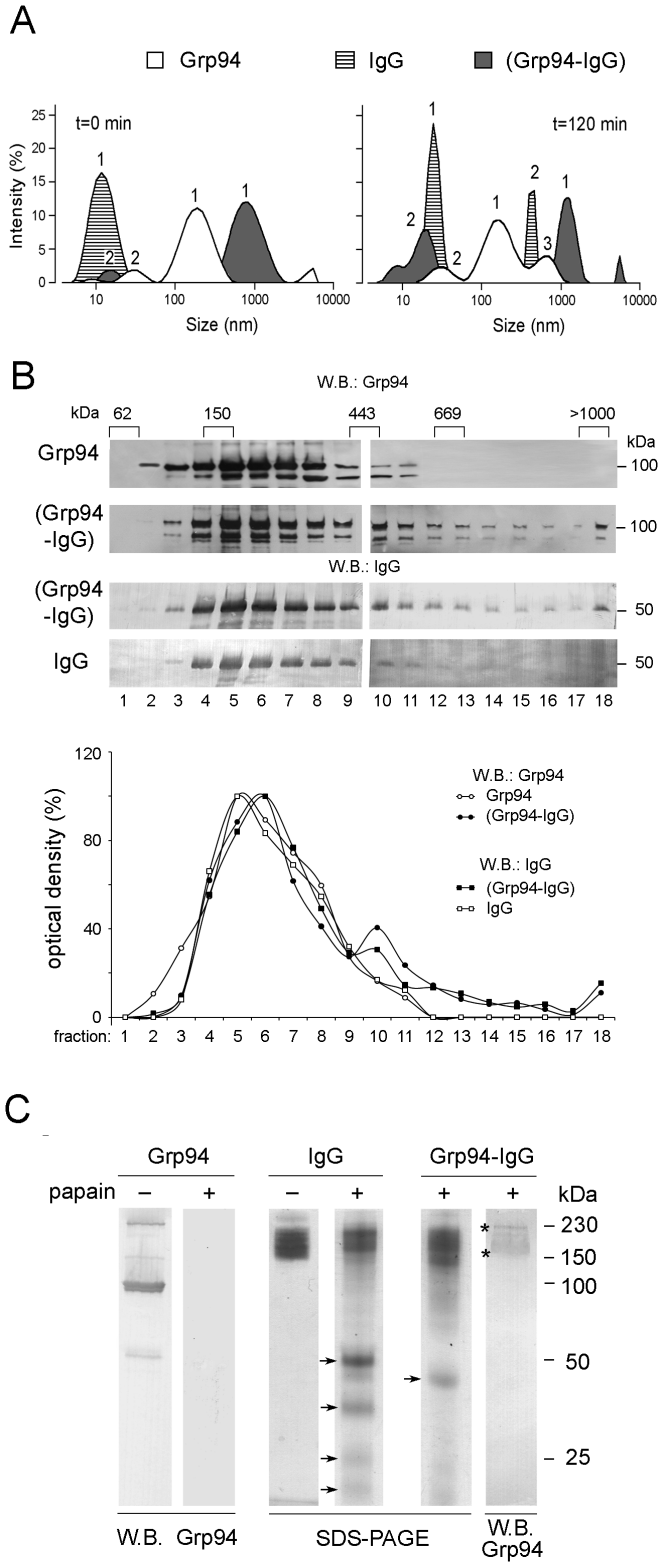
Structural properties of the complex formed by native rat Grp94 with IgG. (**A**) DLS of native Grp94, IgG and Grp94-IgG complex. Spectra represent the frequency distribution of sized species of Grp94 (empty area), IgG (dashed area), and Grp94-IgG complex (gray area). Each peak is the mean of three records, from 14 consecutive measurements over 3 min. Solutions of Grp94 and IgG, both alone and mixed together were analyzed in the cuvette located in the instrument at 37°C (see Methods). Peaks are numbered in decreasing order of intensity of the area. Spectra of a representative experiment of three others made on different occasions are shown. (**B**) Glycerol density gradient centrifugation. Solutions of native Grp94 (0.1 mg/ml) and IgG (0.3 mg/ml), both alone and mixed together after incubation for 120 min, were subjected to fractionation by glycerol density gradient centrifugation (10–40% from fractions 1 to 18) (see Methods). Each fraction (8 µg proteins) was analyzed in SDS-PAGE in reducing conditions and boiling of samples, followed by Western blotting for detecting Grp94 and IgG. Fractions are numbered below blotting. For IgG, only the most representative band at 50 kDa (among the bands that form after reducing treatment of integer IgG) are represented for sake of clarity and uniformity with Grp94 bands. The mass of standard proteins is above the blotting, whereas masses of Grp94 and IgG are on right. The graph below shows the pattern of the optical density of the bands in each fraction expressed as percentage of the maximal intensity (100%) that in each blotting is represented by the fraction with the highest optical density. (**C**) The complex formed with native Grp94 (0.1 mg/ml) and IgG (0.3 mg/ml) after co-incubation at 37°C for 120 min in the presence of ATP was submitted to papain digestion for 8 h at 37°C (see Methods). The extent of proteolysis was followed in SDS-PAGE (10% acrylamide gel) in non-reducing conditions of samples and compared with that of both IgG and Grp94 alone. Western blotting for Grp94 was performed on samples of Grp94 alone and in complex with IgG. 2.5 µg of Grp94 and 7.5 µg IgG were loaded in both control lanes and in the lane of the complex. Arrows indicate the bands of IgG after digestion in both the control and complex. Bands of Grp94 remaining in the bulk of IgG after digestion of the complex are marked by asterisks. On right, molecular masses of reference in kDa.

A direct evidence of the formation of Grp94-IgG complexes was first obtained in experiments of DLS that permits to evaluate the formation of new species by measuring the size distribution of reactants both singularly and after they are mixed together. To this aim, freshly prepared solutions of native Grp94 (0.1 mg/ml) and IgG (0.30 mg/ml) were analyzed both alone and after mixing at 37°C, and the reaction followed for 2 h (Figure 2A). Two main species of control Grp94 were identified in the spectrum, the largest area being referred to the dimer followed in dimensions by the monomer (Figure 2A, peaks 1 and 2, respectively, left panel). The mean size of the main species of Grp94 corresponded to a molecular mass much higher than the expected theoretical value of 200 kDa, justified by the non-globular conformation of the molecule [32]. IgG appeared in a single peak with a mean size consistent with the apparent molecular mass of about 200 kDa (Figure 2A and Table S1 in File S1), whereas the Grp94-IgG complex was easily identified as a distinct entity (peak 1) already evident at time zero, with a mean size four times larger than that of Grp94 dimer (Table S1 in File S1). The almost instantaneous formation of the complex was consistent with the high binding affinity of the reactants, markedly different from the slow rate previously described to occur in Grp94 binding to peptides [15]. After 2 h, both Grp94 and IgG were converted into homo-aggregates of larger sizes, whereas the peak of the complex became sharper, suggesting a dynamic rearrangement of the species characterized by less dispersed forms. Apparently, this was due to the liberation of species of lower sizes, probably IgG, as indicated by the smaller peak partly overlapping that of IgG (Figure 1C and Table S1 in File S1).

The big size of the complex with respect to that of the individual proteins was further confirmed in experiments of glycerol gradient sedimentation analysis (Figure 2B). Both Grp94 and IgG after co-incubation peaked in the same fractions at higher glycerol density, separate from those in which any individual protein was detected. Results were in accord with those obtained in previous experiments [25], in addition revealing the presence of various species of the complex with an exceptionally high mass, as demonstrated by the detection of Grp94 and IgG in the fraction at the highest glycerol density (Figure 2B, far right lane in the blotting).

### Grp94-IgG complexes show an increased resistance to papain digestion

To identify the structural determinants involved in binding, proteolytic digestion of the complex was performed with papain. In preliminary experiments, the papain-to-protein weight ratio of 1∶50 and an 8.0-h incubation at 37°C were proved sufficient to obtain a discrete digestion of control IgG. IgG fragments were visualized in SDS-PAGE (Figure 2C) in a series of bands at molecular masses equal to or lower than 50 kDa, consistent with a mixture of Fab, Fc and subunits of both (arrows). After digestion, the broad band of integer IgG near the cathode (150–230 kDa), albeit still visible, was markedly reduced with respect to that of non-digested IgG. Digestion of the complex yielded a single band at about 40 kDa, not coincident with any of those attributable to proteolytic fragments of control IgG, and a larger band of undigested IgG was also visible at the cathode (Figure 2C). The result was consistent with an increased resistance of the complex to digestion, likely due to masking of proteolytic sites by Grp94. This possibility was supported by results of Western blotting in which Grp94 was detected in the band of integer IgG at the cathode even after papain digestion (Figure 2C, asterisks on the right lane), whereas no positivity for Grp94 was detected after papain treatment of control Grp94 (Figure 2C). However, mass analysis of Grp94-containing bands gave always a negative result for Grp94 (data not shown), and only sequences of IgG were identified, probably due to the overwhelming concentration of IgG in the bands that masked the weaker signal due to Grp94.

### Electron microscopy of Grp94-IgG complexes in absence and presence of ATP

**Figure 3:**
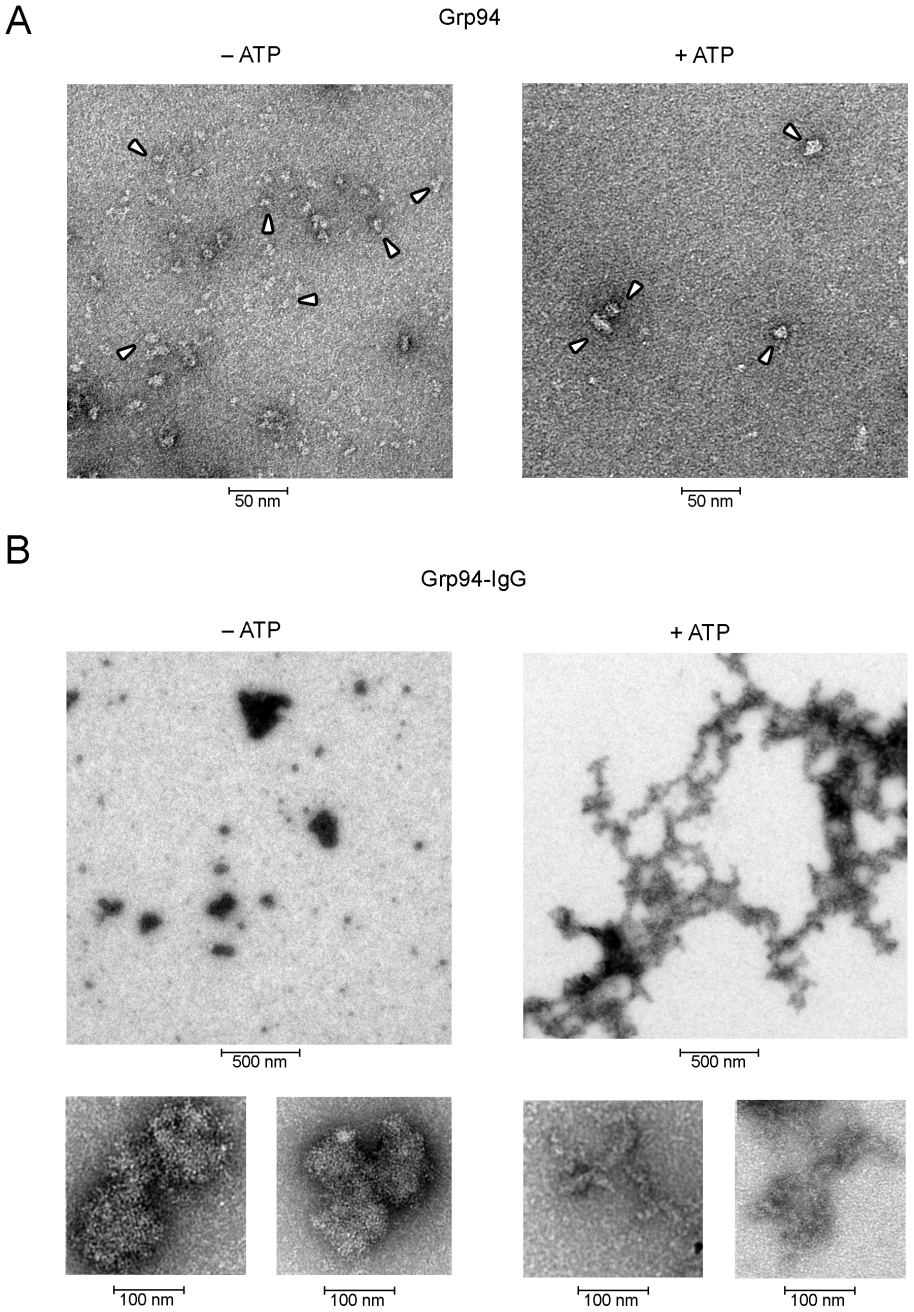
Shape and dimensions of the complex formed by native rat Grp94 with IgG in absence and presence of ATP. (**A**) Representative electron microscopy images of negatively stained native Grp94, treated as specified in Methods, in both absence (control, left panel) and presence of ATP (1.0 mM) (right panel) at the same magnification with a 4.2 Å pixel size. Images are taken from several pictures made on Grp94 solutions (fractions of peak 2 from gel-filtration) in three separate occasions. Arrowheads indicate some of the typical structures of Grp94. (**B**) Representative images of Grp94-IgG complexes obtained by incubating native Grp94 (0.1 mg/ml) with IgG at the Grp94∶IgG molar ratio of 1∶2, in both absence and presence of ATP (1.0 mM) at different magnifications. Images of the complex are obtained from different fields. The larger and smaller boxes have, respectively, 25 Å and 5 Å pixel sizes. Calibration bars are reported below each panel.

A direct evidence of the complex formation was also offered by the electron microscopy (Figure 3A). Pictures of control Grp94 in absence of ATP revealed rod- and ring-like shapes of variable dimensions, whereas in the presence of ATP more compact, ring-shaped structures (of average dimensions of 50–60 nm) were apparent, consistent with the formation of Grp94 oligomers and of higher order aggregates. No difference due to ATP was instead apparent in the pictures of control IgG, and individual IgG molecules were identifiable with the classic Y-like structure of an average length of 20 nm (Figure S2A in File S1, arrows). Striking differences characterized Grp94-IgG complexes in absence or presence of ATP. In the former case, complexes appeared as thick aggregates of globular, irregular shape with a diameter ranging from about 100 to 300 nm (Figure 3B, larger panel on left), closely resembling the high order peptide-Grp94 complexes identified by Linderoth et al. using the scanning transmission electron microscopy [33]. At higher resolutions, Grp94-IgG complexes were resolved as a tight and regular mesh of rod- and Y-like elements organized in tri-dimensional structures (Figure 3B, smaller panels on left). A much more regular structure was visualized in the complex in the presence of ATP, characterized by a flat and elongated network of elements (µm in length) forming geometrical structures in which IgG were partly identifiable at higher resolutions (Figure 3B, panels on right and Figure S2B in File S1). The peculiar shape of the complex, in both absence and presence of ATP, accounted for repeated unsuccessful attempts to obtain crystallization of the complex in any conditions (data not shown), confirming the difficulties encountered also by others to obtain crystallographic models of peptide-Grp94 complexes [33].

### Native rat Grp94 does not form complexes with either one of the IgG subunits

Since experiments of proteolytic digestion of the complex did not permit to assign with certainty the site of Grp94 binding to IgG, whether it pertained to Fab or Fc, we made experiments in which Grp94 was incubated with Fab, Fc and Fab_2_. Fab_2_ were also used to see whether dimeric versus monomeric Fab were required for Grp94 binding to IgG. Native PAGE followed by Western blotting with anti-Grp94 Abs showed that no complex formed in either one of these conditions also in the presence of ATP (Figure S3 in File S1), supporting the conclusion that integer IgG was required to engage a specific binding with Grp94.

### Effect of glycosylation of Grp94 on the binding capacity to IgG

**Figure 4:**
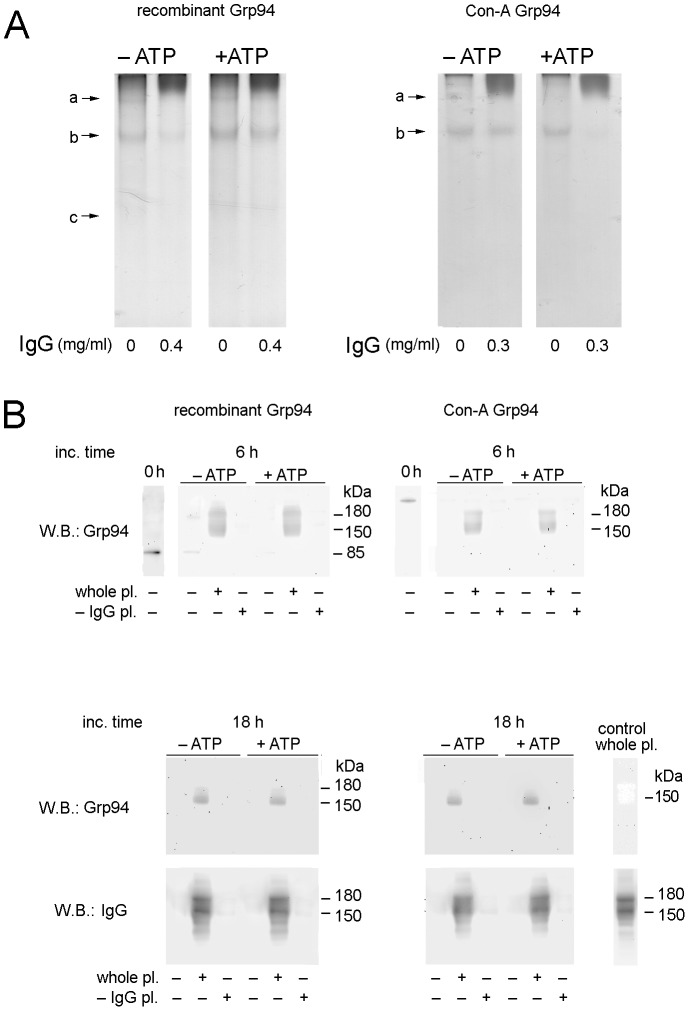
Effects of glycosylation on the property of Grp94 to form complexes with IgG. (**A**) Both recombinant rabbit and Con-A native Grp94 (obtained as specified in Methods) were incubated at the concentration of 0.1 mg/ml in 10 mM Tris HCl (pH = 7.0) both alone and with IgG (Grp94∶IgG molar ratio of 1∶2) for 120 min at 37°C in both absence and presence of ATP (1.0 mM). Three µg of proteins were loaded in each lane and submitted to native PAGE (8% acrylamide, pH = 8.0) and gels stained with Coomassie blue. a, b, c, bands as in Figure 1. (**B**) Both recombinant and Con-A Grp94 (2.5 µg) were incubated with human plasma, both whole and deprived of IgG, as specified in Methods, for 6 and 18 h at 37°C. Three µg of proteins were loaded in each lane and submitted to SDS-PAGE in non-reducing conditions of samples. Lanes of control Grp94 in absence of incubation are on the left of upper panels. Recombinant rabbit Grp94 appears prevalently in its monomeric form, whereas Con-A native Grp94 is visible as a dimer. Bands are shown that are positive for both Grp94 and IgG. Lanes of WB for both Grp94 and IgG in control whole plasma are on right of lower panels.

To address the question of whether glycosylation might influence the binding of Grp94 to IgG, we chose as a negative control recombinant (rabbit) Grp94 expressed in *E. coli* (Figure 4A), and as a positive one native rat Grp94 purified in the last step of purification on the Con-A column to which only the glycosylated form of Grp94 can bind (Con-A Grp94). The recombinant form of rabbit Grp94 available so far is shorter than that of other species (716 residues, 82.5 kDa), lacking the first 79 residues at the N-terminus [28]. However, the alignment with the corresponding sequence of Grp94 of other species (starting from the amino acid 80 of the full length Grp94) shows high degree of homology (96% with human Grp94 and 97% with mouse and rat Grp94) (BLAST, [34]). We thus examined the capacity of both recombinant and Con-A Grp94 to bind to IgG using the Grp94-IgG molar ratio of 1∶2, in absence and presence of ATP, in different incubation conditions.

ATP did not influence at all the capacity of recombinant rabbit Grp94 to form complexes with IgG as judged from native PAGE (Figure 4A, panels on the left: in both absence and presence of ATP, reduction of 100% and 60% of bands a and b, respectively, with respect to the control), whereas ATP favored the binding to IgG of Con-A Grp94 (Figure 4A, panels on right: reduction of 80% of band b with respect to the control). At variance with Con-A Grp94, recombinant Grp94 also showed a higher tendency to oligomerize (band a), especially following heat treatment (42°C for 2 h), a condition that further favored Grp94 binding to IgG (data not shown). Heat treatment instead did not appear to increase further the capacity of Con-A Grp94 to form complexes with IgG (data not shown) with respect to what observed at 37°C in the presence of ATP. To prove that an excess of glycosylation might represent an obstacle to Grp94 to bind IgG in absence of ATP, in incubation experiments with IgG we also used a recombinant form of Grp94 expressed in insect cells (Sf21) known to be highly glycosylated [35]. Similarly to what observed with Con-A Grp94, no complex appeared to form between this glycosylated form of Grp94 and IgG independently of ATP, whereas the addition of ATP partially overwhelmed this negative tendency, causing a 50% reduction in the band of free Grp94, indicative of a limited Grp94-IgG complex formation (Figure S4 in File S1). These results indirectly also suggested that the effect of ATP to induce the complete formation of complexes with IgG of native rat Grp94 in peak 2 from gel filtration (Figure 1C) was likely due to an ATP-dependent conformational change of the glycosylated species of Grp94 present in the population of purified Grp94.

To test the capacity of Grp94 to form complexes with IgG as a function of glycosylation in a setting closer to the *in vivo* conditions, we set up experiments in which recombinant and native Con-A Grp94 were incubated at 37°C at longer incubation times (6 and 18 h) with both whole and IgG-deprived human plasma. Whole plasma would thus represent the medium in which Grp94 that is exposed on the extra-cellular side *in vivo* can encounter circulating IgG at a concentration much higher that those used at the Grp94-to-IgG molar ratios of 1∶2 and 1∶3. As expected, control Grp94, both recombinant and Con-A Grp94, underwent time-dependent degradation, so that after 18 h Grp94 was no longer visible in SDS-PAGE (Figure 4B). However, after incubation with whole, but not IgG-deprived plasma, Grp94 was clearly detectable in bands coincident with those of IgG (Figure 4B), proving that part of Grp94 remained entrapped into IgG even after denaturing conditions of PAGE. After 6 h, positivity was more intense for recombinant Grp94 compared to Con-A Grp94, whereas after 18 h, Grp94 was confined in a single, thinner band with similar intensity in both recombinant and Con-A Grp94. Apparently thus, the higher binding capacity to IgG displayed by non-glycosylated with respect to glycosylated Grp94 seems to be a transient phenomenon occurring early in the first hours of incubation with IgG, whereas in time differences wane off. The lack of any positivity for Grp94 after incubation with IgG-deprived plasma (containing all other proteins, including albumin) supports the specificity of Grp94 binding to IgG, as also confirmed in separate experiments with native rat and rabbit Grp94 incubated with human albumin (Figure S5 in File S1).

### N-terminal domain of Grp94 contains sites of binding to IgG

**Figure 5:**
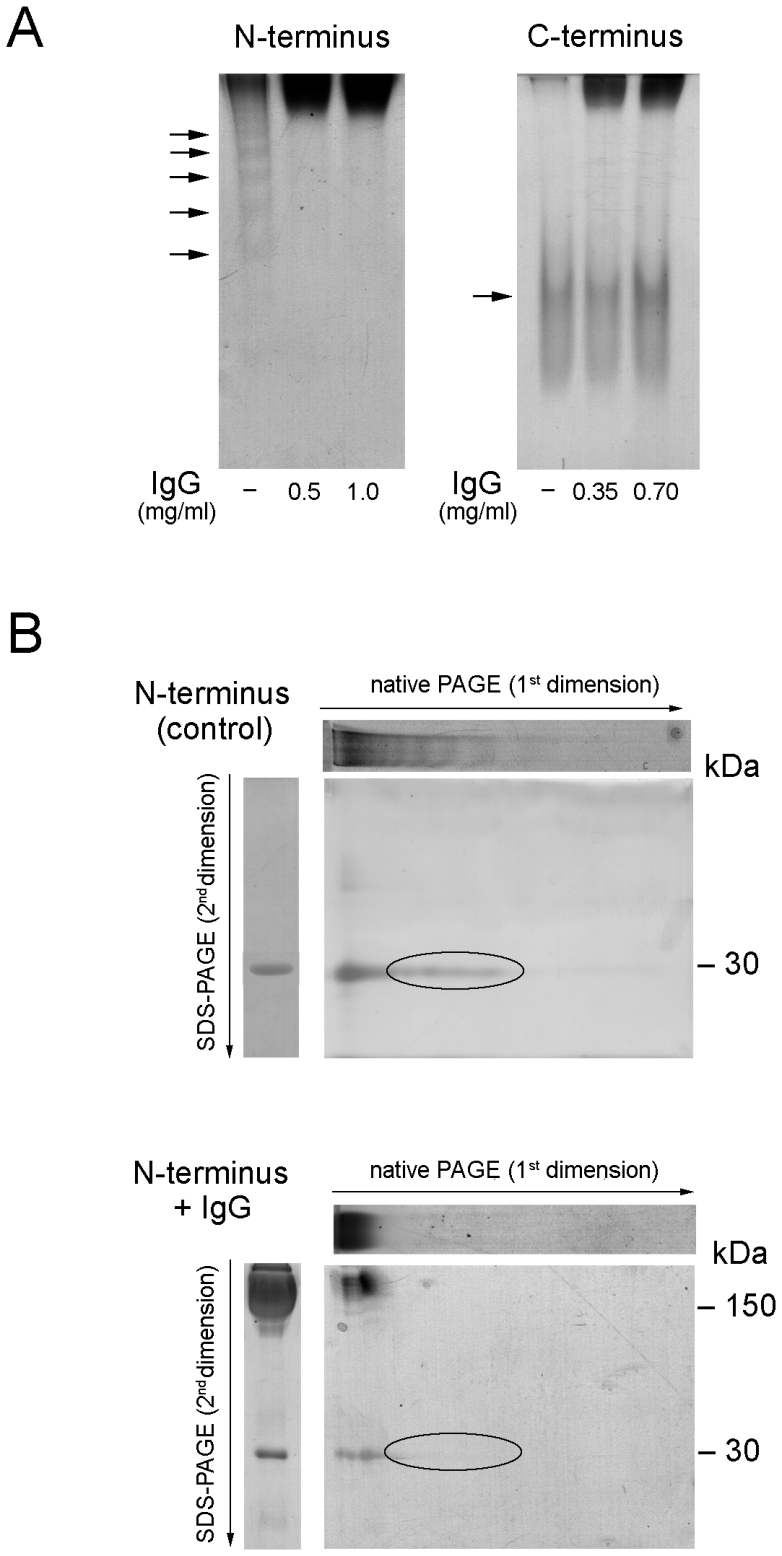
N-terminal domain of Grp94 contains sites of binding to IgG. (**A**) N-terminus and C-terminus of recombinant rabbit Grp94 were prepared as reported in Methods. Each Grp94 fragment (0.1 mg/ml in 10 mM Tris, pH = 7.0) was incubated in absence of ATP for 120 min at 37°C without (control) and with IgG at the indicated concentrations, corresponding to the molar ratios of N- and C-Grp94-to-IgG of 1∶1 and 1∶2 (for the lower and higher IgG concentration, respectively). 2.5 µg Grp94 were loaded and analyzed in SDS- (not shown) and native PAGE. Arrows mark bands of N-Grp94 and of C-Grp94. (**B**) 2D PAGE of N-Grp94 incubated both alone and with IgG at 0.5 mg/ml. Above the gel and on left, are lanes of reference from the first dimension PAGE (8% acrylamide, pH = 8.0) and from control SDS-PAGE (10% acrylamide, in non-reducing conditions). On right, the molecular mass of reference in kDa. In both gels, circled bands are those that disappear following incubation with IgG.

To establish whether the binding to IgG involved N- or C-terminal domains of Grp94, we took advantage of recombinant N- and C-terminal fragments of rabbit Grp94 expressed in *E. coli* to perform experiments of incubation with IgG. N-terminal fragment (N-Grp94) of 30 kDa (corresponding to amino acid residues 80-309 in the sequence of whole human and rat Grp94) formed a ladder of bands in native PAGE (Figure 5A, left panel, arrows), suggesting the assembly of the fragment in forms of different masses and charge density (dimers and higher-order complexes). A similar finding has been reported with N-terminus of murine recombinant Grp94, the different mobility of which in native PAGE was attributed to different conformations of the same sized species [15]. When resolved in the second dimension, the ladder of bands collapsed into that of the monomer, whereas dimer was only weakly stained (Figure 5B, upper panel), proving that different conformers of N-Grp94 existed in solution. Following incubation with IgG at the N-Grp94-to-IgG molar ratio of 1∶1 and mostly 1∶2 (0.5 and 1.0 mg/ml IgG, respectively), bands of N-Grp94 disappeared, being apparently sequestered within the IgG band at the cathode (Figure 5A, left panel). In the second dimension PAGE this band was resolved exclusively as monomer, much less stained compared with its own control (Figure 5B, lower panel). The addition of ATP did not modify the electrophoretic pattern of the complex, similarly to what observed with full-length rabbit Grp94, implying that N-Grp94 was already in the conformation suitable for binding to IgG.

To explore the binding capacity of the C-terminal recombinant Grp94 (C-Grp94) to IgG, we used the fragment of 42 kDa corresponding to amino acid residues 495-803 in the sequence of whole human and rat Grp94. In SDS-PAGE, C-Grp94 was present in the form of monomer, dimer and higher-order aggregates reduced to the monomer with β-mercaptoethanol (data not shown). In native PAGE (Figure 5A, right panel), C-Grp94 focused in a single diffuse band that did not change mobility after incubation with IgG thus negating any capacity to bind to IgG.

## Discussion

In this work we wanted to investigate some still unanswered questions of pathophysiological relevance about the nature and characteristics of complexes that Grp94 can form with IgG in pathological conditions in which Grp94 is liberated in the extracellular space where it is always sensed as immunological and inflammatory danger [17], [18]. IgG are amongst the few known client proteins of ER-resident Grp94 [16], [36], and the function of Grp94 is to bind a more mature form of IgG, performing the correct assembly of the IgG molecule before its secretion from the cell [37]. However, the possibility that Grp94 can bind to IgG in a manner different from that predicted from its chaperoning function has been raised in a previous work in which complexes have been observed to form between Grp94 and human non-immune IgG *in vitro* [25]. Such complexes in addition turned out to be functionally similar to those identified in the plasma of diabetic subjects in whom they drive a high risk of vascular damage [21], [24].

Our present work confirms and extends previous results [25], offering a series of experimental proofs in favor of the elevated specificity and stability of the binding that Grp94 forms with IgG, also identifying the portion of the molecule, in both Grp94 and IgG, involved in binding. Since Grp94 found in complexes isolated from plasma of diabetic subjects was apparently non-glycosylated [20], this prompted us to investigate the influence that glycosylation of Grp94 might have on the capacity to bind to and forming complexes with IgG. It is known that heterogeneous forms of Grp94 exist in nature and differences are often attributable to post-translational modifications that affect charge and size of the protein [35], [38], [39]. However, the functional meaning of the different Grp94 species, in particular those glycosylated, in relation to the extra-cellular presentation of Grp94 and its immunogenicity is still an open, debated question [38], [40]. In the purification process of native Grp94 that included gel filtration chromatography in the final step, the pure form of Grp94 (in peak 2) turned out to be a mixture of glycosylated and mostly non-glycosylated species, as specified thereafter below and also revealed by specific staining of gels for glycoproteins (data not shown). The most part of this pure Grp94 was able to bind rapidly to IgG, forming complexes of elevated dimensions and stability, as demonstrated by experiments of glycerol density gradient centrifugation and light scattering (Figure 2). The almost instantaneous formation of the complex testified the high affinity of binding, a property that has not been described so far for Grp94 binding to any protein and peptides [15]. To prove that the species of Grp94 specifically and rapidly engaged in binding to IgG was non-glycosylated, or at least only weakly glycosylated, whereas glycosylation could represent an obstacle to binding, we used different experimental approaches. First, we observed that the addition of ATP to the species of native Grp94 that was apparently refractory to bind to IgG induced the formation of Grp94-IgG complexes in which every Grp94 species was involved in binding (Figure 1C and D). Since Grp94 adopts different conformations following ATP binding [6], [11], [30], [31], and since ATP binding sites in Grp94 are located in the N-terminal domain nearby the canonical glycosylation site (Swiss-Prot Data Bank, P41148) [39], results could be reasonably explained by admitting that Grp94 refractory to binding was glycosylated and that ATP induced in the glycosylated species of Grp94 a conformation that rendered available the sites of binding previously masked by oligosaccharide residues. The effect of ATP was apparently associated with an increased aggregating tendency of Grp94 (Figure 1E), a condition that in turn can enhance the chaperoning activity of Grp94 [10], [41]. We actually observed that in the presence of ATP native Grp94 assumed a more compact structure and formed complexes with IgG with a geometrical configuration and dimensions strikingly different from the unordered shapes assumed by complexes in absence of ATP (Figure 3). The pictures at the electron microscopy were consistent with the possibility that oligomerization of Grp94 caused the exposure and spatial assembly of the binding sites as to increase the rate of binding to IgG and favor the inter-molecular aggregation of IgG. The complexity of the structure in the presence of ATP can justify the high stability of the complex evidenced also in the resistance to the proteolytic digestion by papain (Figure 2).

Another approach to the comprehension of the influence of glycosylation on Grp94 binding capacity to IgG included the use of fully glycosylated and non-glycosylated species of Grp94 as a positive and negative control, respectively. The former was obtained by passing native Grp94 in the final step of purification into Con-A column to obtain only glycosylated forms, whereas the latter was rabbit Grp94 expressed in *E. coli*. It is a common knowledge that non-glycosylated proteins show a higher tendency to aggregate whereas glycosylation favors solubility and stabilizes the structure of the protein conferring on it a higher resistance to the proteolytic degradation [42]. While glycosylated Con-A Grp94 as such was refractory to bind IgG in the common incubation medium, and ATP was necessary to counteract this negative effect, recombinant Grp94 did not need ATP to form complexes with IgG (Figure 4A), in addition displaying a higher tendency to oligomerize especially after heat treatment. Apparently thus, absence of glycosylation in Grp94 was associated with a conformation suitable for binding, similarly to what observed with ATP on glycosylated species of native Grp94. In support of this conclusion were also results of experiments performed with recombinant Grp94 obtained in insect cells (Sf1) known to be highly glycosylated [35]. This Grp94 was completely unable to form complexes with IgG and only the addition of ATP partially overwhelmed the inhibitory effect of the extensive glycosylation (Figure S4 in File S1). Overall, these results indicated that the capacity of Grp94 to bind to IgG was independent of both the species from which Grp94 was obtained and the nature of the protein, whether native or recombinant, post-translational modifications in glycosylation being instead crucial in determining accessibility to binding sites.

We then wanted to verify whether the conclusions drawn from experiments with differently glycosylated species of Grp94 could also be extended to the *in vivo* conditions as those identified in the plasma of diabetic subjects [20], [21], [24] in which Grp94 is present in complexes with IgG. To this aim, we set up experiments in which both glycosylated Con-A and recombinant rabbit non-glycosylated Grp94 were incubated with both whole and IgG-deprived human plasma at longer incubation times, representing a setting closer to physiological conditions (Figure 4B). Results of these experiments unequivocally demonstrated that Grp94 remained linked to plasma IgG even after 18 h incubation, whereas no Grp94 was detected in plasma deprived of IgG that contained all other proteins. This proved the specificity of Grp94 binding to IgG, excluding the possibility that Grp94 could bind to other plasma proteins, in particular albumin, as also demonstrated in separate experiments of incubation with human serum albumin (Figure S5 in File S1). It was also apparent that the difference in binding capacity to IgG observed between the non-glycosylated and glycosylated Grp94 in the *in vitro* incubation medium, was not so significant when incubation was conducted in the physiological medium of plasma, especially after longer incubation times, whereas a higher binding capacity was still displayed by non-glycosylated Grp94 at shorter incubation times (Figure 4B). Thus, although absence of glycosylation favors a rapid and stable binding of Grp94 to IgG, glycosylation appears to represent a temporary obstacle to binding that can be overcome by ATP or even disappears independently of ATP when incubation is protracted for longer times in a more physiological setting. The finding that in these experimental conditions Grp94 was almost exclusively present in bands coincident with those of IgG but not at higher molecular masses (Figure 4B) is consistent with the observation that Grp94-IgG complexes undergo a time-dependent dynamic rearrangement (Figure 2) so that in denaturing conditions of electrophoresis only the fragments of Grp94 that remain closely associated with IgG can be evidenced in Western blotting. It is of interest to note that this picture is strikingly similar to those obtained in *ex-vivo* experiments on plasma of diabetic subjects in which Grp94 was exclusively found inextricably linked to IgG in bands with a mass corresponding to that of IgG [20], [24].

Our results also offer the experimental proof showing that the N-domain of Grp94 contains the sites for binding to IgG, whereas the C-terminal portion is excluded (Figure 5). The N-terminus fragment of recombinant rabbit Grp94 was indeed able to bind to IgG in absence of ATP, as did the full-length Grp94, a result in accord with previous studies demonstrating the capacity of the recombinant N-terminal fragment of Grp94 to bind peptides [15] also in absence of ATP [43]. We cannot establish what residue(s) in the N-domain of Grp94 are specifically involved in the complex formation, although the permissive effect of ATP in favoring binding to IgG of glycosylated Grp94 suggests that sites of binding to IgG are distinct from the nucleotide binding sites. The finding that neither Fab, nor Fab_2_ and Fc were singularly able to form complexes with Grp94, as the whole IgG molecule instead did, strongly supports the involvement of the hinge region in the binding, a conclusion also indirectly based on results of proteolytic digestion of the complex (Figure 2). Altogether our results would support a mechanism of binding in which the smaller unit of the complex is given by a dimer of Grp94 and two whole IgG molecules that engage binding with sites on the outer side of each N-terminal domain of the dimer. The possibility that binding to IgG can occur in the inside surface of the cavity formed by N-terminal domains, as proposed for other client proteins of Grp94 [31], is negated by the steric hindrance of the IgG molecule that cannot accommodate the internal cavity of Grp94 dimer.

In conclusion, our work shed light on the structural properties of the complex that Grp94 forms with human IgG, offering the experimental evidence to support the elevated specificity and stability of the complex, with particular regard to the role that a variable extent of glycosylation might have on the binding capacity of Grp94. Results have relevance for the *in vivo* pathological conditions in which Grp94 is presented in the extra-cellular setting to circulating IgG. The rapidity with which Grp94 binds to IgG and the extraordinary stability of the complex demonstrated *in vitro* predict the immune/inflammatory response that might follow the formation of such complexes *in vivo*.

## Supporting Information

File S1 **Combined supporting information, containing: Materials S1, Methods S1, Methods S2, Table S1, Figure S1, Figure S2, Figure S3, Figure S4, Figure S5.**
(PDF)
