# Supplementary material for: Structural Insights into Complexes of Glucose-Regulated Protein94 (Grp94) with Human Immunoglobulin G. Relevance for Grp94-IgG Complexes that Form In Vivo in Pathological Conditions
Source: PLoS One. 2014 Jan 28;9(1):e86198. doi: 10.1371/journal.pone.0086198 (PMC3904872; doi:10.1371/journal.pone.0086198)
Supplement: File S1 — Combined supporting information, containing: Materials S1, Methods S1, Methods S2, Table S1, Figure S1, Figure S2, Figure S3, Figure S4, Figure S5. (PDF) [file pone.0086198.s001.pdf]

Supporting Information for:

Structural insights into complexes of Glucose-regulated Protein94 (Grp94) with human immunoglobulin G. Relevance for Grp94-IgG complexes that form *in vivo* in pathological conditions

Table of contents:

Materials S1	Pag. 2
Methods S1	Pag. 2
Methods S2	Pag. 3
Table S1	Pag. 3
Figure S1	Pag. 4
Figure S2	Pag. 5
Figure S3	Pag. 6
Figure S4	Pag. 7
Figure S5	Pag. 8

Materials S1

Coomassie brilliant blue G, acrylamide-bis acrylamide, ATP, glycine, Trizma-base, HEPES, SDS, papain (crystallized, from Papaya latex) and human serum albumin (HSA) were from Sigma-Aldrich (St. Louis, MO, USA). Acetic acid, formaldehyde solution, methyl and ethyl alcohol and ammonia solution were from Carlo Erba (Milan, Italy). DTT was from Invitrogen (Grand Island, NY, USA). Heparin Hi-trap column was from Amersham Bioscience (Uppsala, Sweden) and PVDF membrane from Millipore (Billerica, MA, USA).

Methods S1

Purification of native Grp94

To purify Grp94, the liver of 10 rats was minced and homogenized at +4° C (5,000-10,000 r.p.m. for 40-80 s with a Polytron Homogenizer, Kinematica, Lucerne, Switzerland) in the homogenization buffer with 50 mM Tris-HCl pH=7.5, 5.0 mM EDTA, 1 mM EGTA, 0.1 mM sodium orthovanadate, 0.25 M sucrose and 0.01 mM PMSF. The homogenate was centrifuged at 10,000x*g* for 10 min, the precipitate discarded and the supernatant centrifuged at 105,000x*g* for 1 h. The pellet (microsomal fraction) was solubilized in the buffer containing 25 mM HEPES (pH=7.0), 10% glycerol, 1% Triton X-100, 1 mM EDTA and 100 µM PMSF, then stirred for 2 h at +4° C followed by centrifugation at l00,000x*g* for 1 h. The insoluble material was discarded and the supernatant subjected to a DEAE-Sepharose column (Pharmacia, Uppsala, Sweden) previously equilibrated with buffer A (25 mM HEPES, pH=7.0, containing 10% glycerol, 0.1 % Triton X-100, 1 mM EDTA, and 100 pM PMSF). Proteins eluted with a linear gradient of NaCl (from 0 to 0.6 M) in buffer A and fractions collected by monitoring the absorbance at 280 nm and assayed for immuno-reactivity to Grp94. Grp94 eluted from DEAE-Sepharose column with 0.4 M NaCl. Grp94-containing fractions were pooled and submitted to a Heparin-Sepharose column equilibrated with buffer A. A linear gradient of NaCl (from 0 to 0.8 M) in buffer A was used for protein elution, and Grp94 was detected in fractions eluting at 0.5 M NaCl. Grp94 fractions were then chromatographed on a HiLoad 26/60 Superdex 200 prep-grade column (26 mm×600 mm, Pharmacia), previously equilibrated with buffer B (20 mM Tris-HCl, pH=7.5, 1 mM EDTA, 1 mM EGTA, 10% glycerol, 0.05 mM PMSF) containing 0.5 M NaCl. Elution was made at the flow rate of 0.75 ml/min, and fractions (1.5 ml each) every four were tested for Grp94 in immunoblotting with anti-Grp94 Abs.

Methods S2

Recombinant Grp94

Recombinant polypeptides corresponding to different regions of Grp94 were prepared after sub-cloning and transformation in the expression vector pQE (Qiagen, Venlo, The Netherlands) of restriction fragments of rabbit Grp94 cDNA. The almost complete recombinant Grp94 protein was obtained from the whole insert of the rabbit Grp94 cDNA clone 2.52 and corresponded to protein residues 107-803 with the poly-histidine sequence MRGSHHHHHHGSGCRNSARA added at the N-terminus for purification [1].
The polypeptide 2.5 was obtained from the EcoRI-PstI fragment of the rabbit Grp94 cDNA clone 2.5 and corresponded to protein residues 80-309 (numbered including the N-terminal signal peptide). The poly-histidine sequence MRGSHHHHHHGIRMR_ ARYPGSTCRNSARA was added at the N-terminus for purification [1].
Polypeptide 7B derived from the HindIII-BamHI fragment of the rabbit Grp94 cDNA clone 2.52 and corresponded to protein residues 495-803. The poly-histidine sequence MRGSHHHHHHGSACELGTPGRPAAKL was added at the N-terminus for purification.

References

1. Frasson M, Vitadello M, Brunati AM, La Rocca N, Tibaldi E et al. (2009) Grp94 is Tyr phosphorylated by Fyn in the lumen of the endoplasmic reticulum and traslocates to Golgi in differentiating myoblasts. Biochim Biophys Acta 1793: 239-252.

Table S1.
Data of peaks recorded in light-scattering.
Data refer to peaks shown in Figure 2A. Numbers for any parameter are mean (± SD) of values of three records each of 14 consecutive measurements on sample solutions at 37° C. Solutions were tested both fresh (t=0) and after 2 h incubation at 37° C. Intensity of the area represents the relative intensity (%) calculated for each peak on the total area of the species in any sample. An intensity equal to or below 5% is omitted as not significant.

		**Peak 1**		**Peak 2**		**Peak 3**	
incubation time (min)	sample	area intensity (%)	mean size (nm)	area intensity (%)	mean size (nm)	area intensity (%)	mean size (nm)
0	Grp94	86.7 ± 3.5	202.0 ± 15.2	8.0 ± 1.8	21.3 ± 2.8	–	–
	(Grp94–IgG)	87.5 ± 5.1	886.1 ± 10.6	10.1 ± 1.4	17.2 ± 1.0	–	–
	IgG	99.7 ± 0.6	12.9 ± 0.3	–	–	–	–
120	Grp94	70.4 ± 6.9	194.2 ± 47.6	19.3 ± 4.7	29.0 ± 7.9	9.4 ± 2.6	605.1 ± 53.7
	(Grp94–IgG)	51.0 ± 5.7	1283 ± 97.5	41.9 ± 3.5	17.5 ± 0.8	–	–
	IgG	76.6 ± 5.5	25.0 ± 0.4	23.4 ± 5.5	359.5 ± 95	–	–

Figures S1

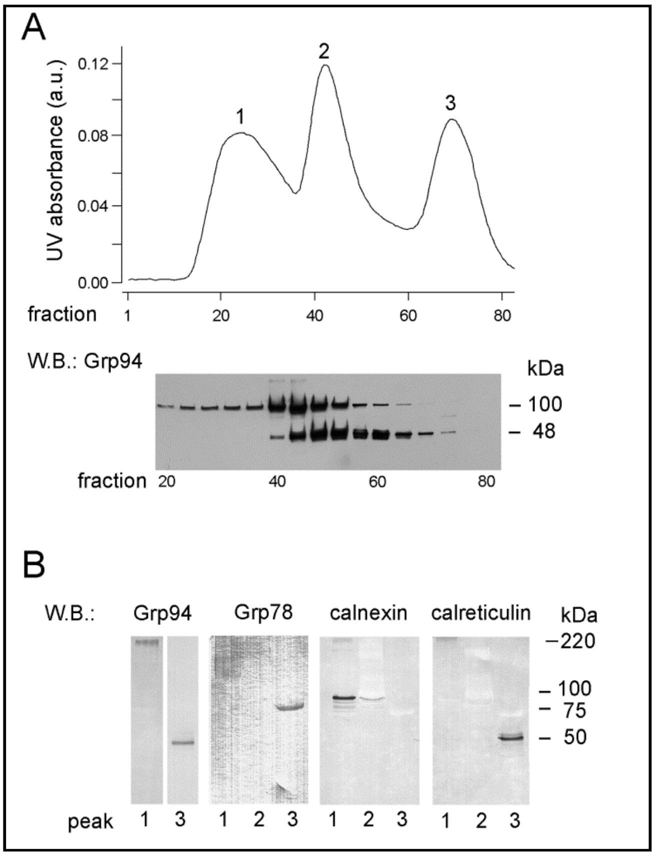

Figure S1. **Characterization of native Grp94**. (**A**) Elution pattern of Grp94 from the Superdex-200 column in the final step of purification of rat Grp94 from hepatocyte microsomes. A representative chromatogram is shown of four separate purification procedures. Elution was monitored by UV detection at 280 nm and proteins collected in 90 fractions of 1.5 ml each. Fifty μl of fractions from 20 to 36 (peak 1), 37-56 (peak 2) and 57-80 (peak 3) were loaded every four in SDS-PAGE (10% polyacrylamide gel, under reducing conditions) and probed in Western blotting with rabbit polyclonal anti-Grp94 Abs (below the elution pattern). Grp94 in peak 1 was exclusively in form of aggregates disrupted by SDS, whereas peak 3 contained the C-terminal fragment of Grp94. Molecular mass of the two main species of Grp94 are on right. (**B**) Western blotting of the indicated proteins on representative fractions of the elution peaks after SDS-PAGE under non-reducing conditions of samples. Three μg proteins were loaded in each lane. Monoclonal Abs were used for detecting Grp94. On right, molecular masses of reference in kDa.

Figure S2

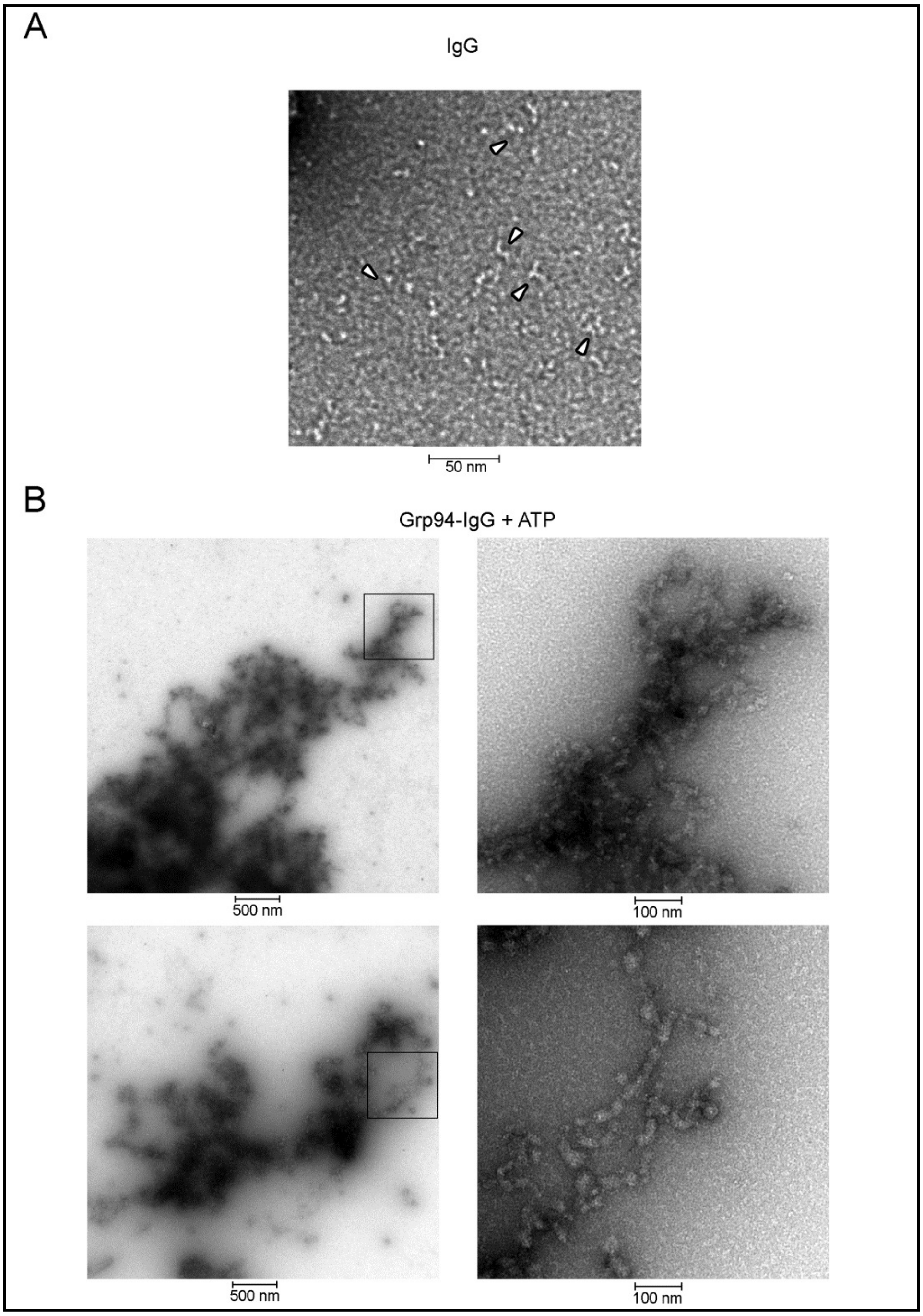

Figure S2. **Electron microscopy of IgG and of Grp94-IgG complexes.** (**A**) A representative image of control human IgG at 3.6 Å pixel size, showing typical Y-shaped forms (arrowheads). (**B**) Images of the complex formed with native rat Grp94 and IgG incubated in the presence of ATP (1.0 mM). On left, Grp94-IgG complex images at very low magnification with a 40 Å pixel size that permits to appreciate the complexity of Grp94-IgG network. On right, a 4x magnification of the squared area in the corresponding left images (10 Å pixel size), revealing at a higher definition the intricate aggregation of elements forming the complex. The calibration bar below each image.

Figure S3

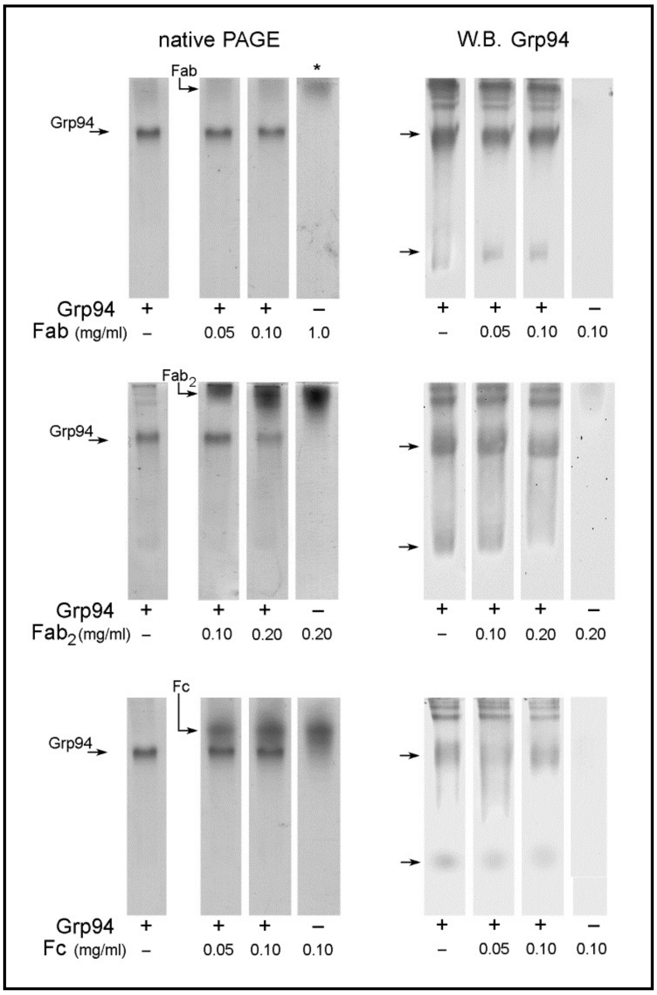

Figure S3. Fab, F(ab)$_2$ and Fc do not form complexes with Grp94. Native PAGE (left panels) and corresponding Western blotting with anti-Grp94 monoclonal Abs (right panels) of 30 µl (3.0 µg) of native rat Grp94 (representative fractions of peak 2 from gel-filtration,) were incubated both alone (control) and with Fab, F(ab)$_2$ and Fc fragments (at the indicated concentrations) with the addition of 1.0 mM ATP (see Methods). In separate lanes, the highest amount of Fab, F(ab)$_2$ (6.0 µg) and Fc (3.0 µg) were also loaded as further control. Control Fab were loaded at 10 µg (asterisked lane), since even at the highest concentration used with Grp94, Fab was not visualized by Coomassie blue staining.

Figure S4

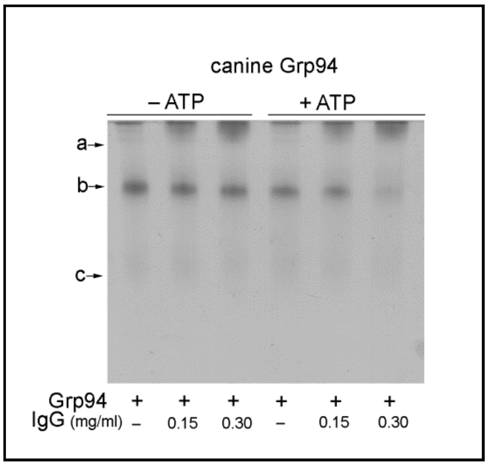

***Figure S4*. Highly glycosylated Grp94 does not form complexes with IgG in absence of ATP.** A purified recombinant form of canine Grp94 expressed in insect cells (Sf21) was purchased from Enzo Life Sciences (Inc. Farmingdale, NY) and used in incubation experiments with IgG at the same concentration (0.1 mg/ml) used with native rat Grp94 (see also legend to Figure 1B) in both absence and presence of ATP (1.0 mM). After 120 min incubation at 37 °C, samples were processed in native PAGE and the complex formation with IgG evaluated by the disappearance of the band of Grp94 (arrow). A 50% reduction of Grp94 band is calculated by densitometric analysis after Grp94 was incubated at the 1:2 molar ratio with IgG in the presence of ATP, indicative of a partial formation of the complex.

Figure S5

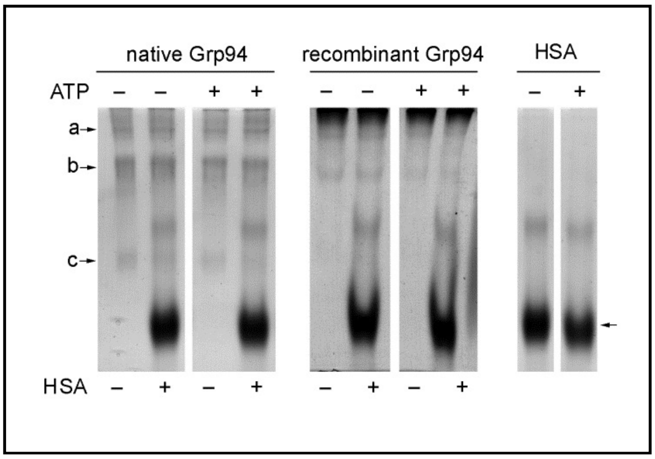

Figure S5. Grp94 does not form complexes with human serum albumin. Native rat Grp94 (from peak 2 of gel filtration) and recombinant rabbit Grp94 (both 0.1 mg/ml in 10 mM Tris-HCl, pH 7.0) were incubated both alone (control) and with HSA at the final molar ratio of 1:2 (0.12 mg/ml and 0.13 mg/ml of HSA for rat and rabbit Grp94, respectively) for 120 min at 37 °C, in both absence and presence of ATP (1.0 mM). Samples were processed in native PAGE (as reported in the legend to Figure 1B). Lanes of control HSA used at the highest concentration in the incubation with Grp94 are on right. Arrows indicate the bands of Grp94 as in Figures 1 and 4. The monomeric form of HSA is shown in the heavily stained band running to the anode due to the negative charges.
